# Supplementary material for: Tilapia Lake Virus Does Not Hemagglutinate Avian and Piscine Erythrocytes and NH4Cl Does Not Inhibit Viral Replication In Vitro
Source: Viruses. 2019 Dec 12;11(12):1152. doi: 10.3390/v11121152 (PMC6950307; doi:10.3390/v11121152)
Supplement: Supplementary file 1 [file viruses-11-01152-s001.zip › viruses-641471-SI.pdf]

SUPPLEMENTARY MATERIAL

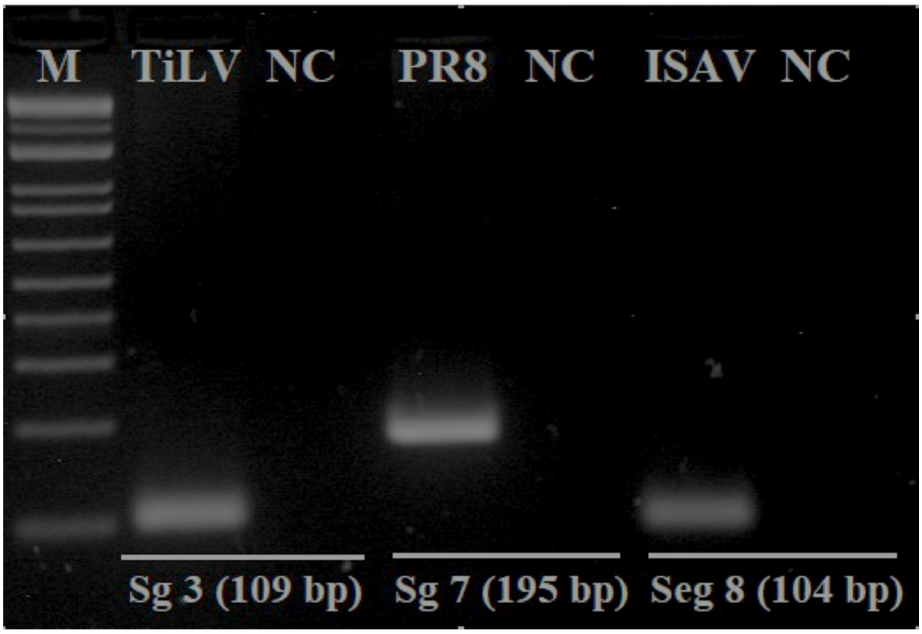

Figure S1. Gel electrophoresis verification of the viruses used for hemagglutination assay. Lane M: DNA ladder (Marker); lane TiLV: Tilapia lake virus, lane PR8: Influenza virus A PR8; lane ISAV: Infectious salmon anemia virus and lane NC: Negative control (RNase-free water).

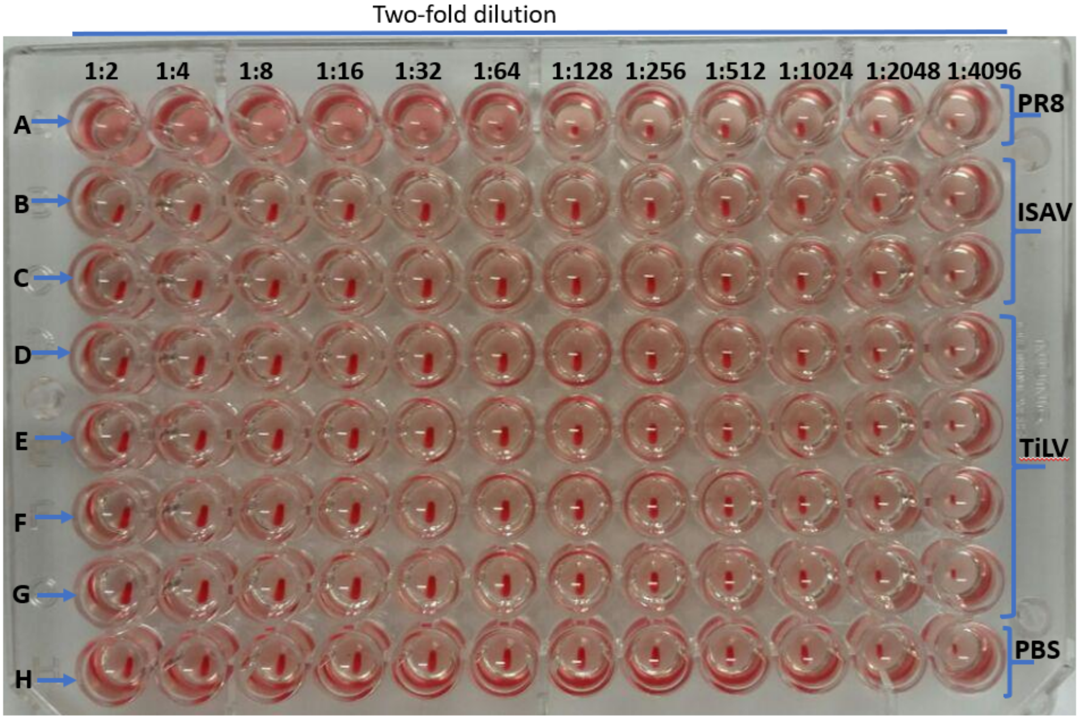

Figure S2. Hemagglutination of turkey red blood cells by influenza PR8 (512HA/50µl), ISAV (10^6 $TCID_{50}$/mL), TiLV (10^7, 10^6, 10^5 and 10^4 $TCID_{50}$/mL wells D to G, respectively) after 1 h incubation at room temperature. Stripping of turkey RBCs in a hemagglutination plate tilted at 60 degrees angle.

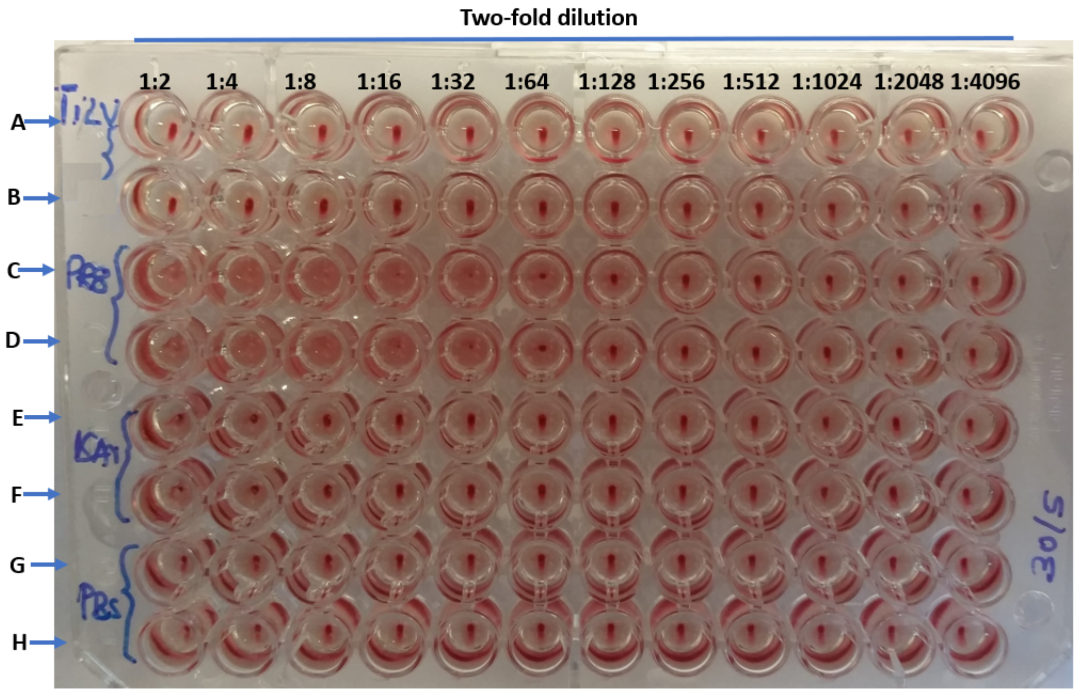

Figure S3. Hemagglutination of tilapia red blood cells by Influenza PR8 (512HA/50µl), ISAV (10^6 $TCID_{50}$/mL), and TiLV (10^7 $TCID_{50}$/mL) after 1 h incubation at room temperature. Stripping of tilapia RBCs in a hemagglutination plate tilted at 60 degrees angle.

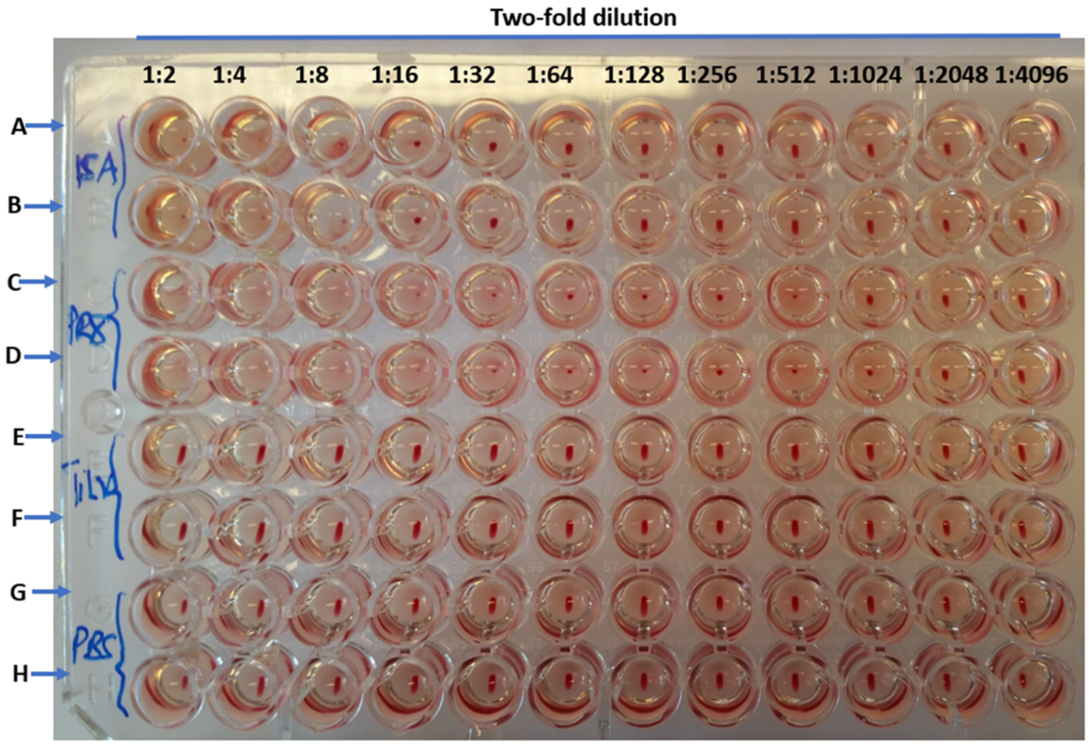

Figure S4. Hemagglutination assay of Atlantic salmon red blood cells by Influenza PR8 (512HA/50µl), ISAV (10^6 $TCID_{50}$/mL), and TiLV (10^7 $TCID_{50}$/mL) after 1 h incubation at room temperature. Stripping of Atlantic salmon RBCs in a hemagglutination plate tilted at 60 degrees angle.

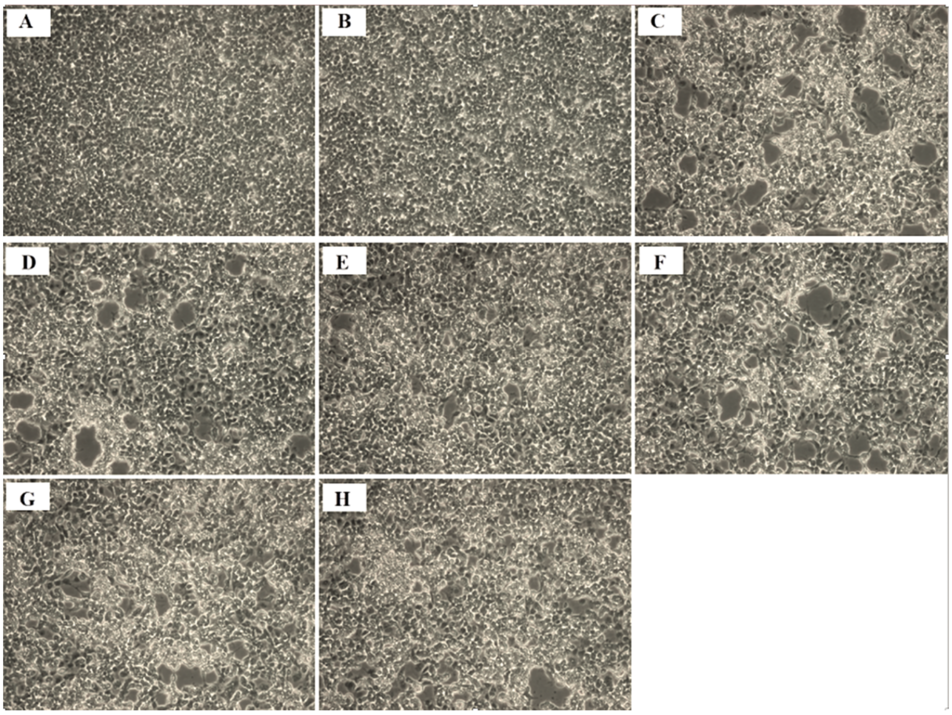

Figure S5. Ammonium chloride treatment of E-11 cells infected by TiLV at 0, 1, 2 and 3 hours post infection. Replication inhibition of TiLV in E-11 using 30 mM of Ammonium chloride (NH_4Cl) 3 dpi (10X magnification). **Figure 5A**: Negative control (untreated cells), **Figure 5B:** Negative control (treated cells), **Figure 5C:** Positive control, **Figure 5D:** NH_4Cl added 30 minutes before infection, **Figure 5E:** NH_4Cl added during adsorption, **Figure 5F:** NH_4Cl added 1 hpi, **Figure 5G:** NH_4Cl added 2 hpi and **Figure 5H:** NH_4Cl added 3 hpi.
